# beta-estradiol attenuates the anti-HIV-1 efficacy of Stavudine (D4T) in primary PBL

**DOI:** 10.1186/1742-4690-5-82

**Published:** 2008-09-22

**Authors:** Mingjie Zhang, Qingsheng Huang, Yong Huang, Owen Wood, Weishi Yuan, Caren Chancey, Sylvester Daniel, Maria Rios, Indira Hewlett, Kathleen A Clouse, Andrew I Dayton

**Affiliations:** 1 Center for Biologics Evaluation and Research, Food and Drug Administration, 1401 Rockville Pike, Rockville, MD 20852, USA; 2 Center for Drug Eveluation and Research, Food and Drug Administration, 5600 Fishers Lane, Rockville, MD 20857, USA

## Abstract

**Background:**

Female hormones are known to play an important role in predisposition for many infectious diseases. Recent work suggests there are gender effects in HIV/AIDS progression. Here we ask whether the sex steroid hormone β-estradiol affects the replication of HIV-1 or the efficacy of a common anti-retroviral drug, Stavudine (D4T).

**Results:**

Human PBL were infected with HIV-1 in the presence or absence of combinations of sex steroid hormones and the anti-retroviral drug, D4T. After seven days in culture, viral supernatants were assayed for HIV-1 p24 protein. β-estradiol resulted in a modest inhibition of HIV-1 replication of ~26%. However, 2 nM β-estradiol increased the amount of HIV-1 replication in the presence of 50 nM D4T from a baseline of 33% (+/- SE = 5.4) to 74% (+/- SE = 5.4) of control virus levels in the absence of drug. Both results were statistically highly significant (p < 0.001). β-estradiol did not increase the replication of a D4T-resistant strain of HIV in the presence of D4T. The effects were unlikely to be due to general cell inhibition or toxicity because these concentrations of drug and hormone cause no cytotoxicity in PBL as measured by trypan blue exclusion.

**Conclusion:**

β-estradiol inhibited both HIV-1 replication in primary human PBL and the antiretroviral efficacy of D4T in PBL cultures. To optimize antiretroviral drug therapy, it may be necessary to monitor patient hormonal status.

## Background

Although there is evidence that viral load and anti-retroviral responses of women differ from those of men [1-3], little is known about gender-specific effects of HIV infection and treatments. Female hormones, including hormonal contraceptives, are known to play an important role in predisposition for many infectious diseases [4]. Whether sex steroid hormones influence susceptibility to HIV-1 infection, severity of symptoms, risk of disease progression or interference of anti-retroviral therapy is not clear. However, a recent epidemiology study reported that the HIV-1 viral load in blood is lower in women than in men at similar stages of HIV-1 infection, suggesting that there are gender effects in HIV/AIDS progression [5]. Furthermore, Lee et al reported that progesterone and Zidovudine (AZT) synergistically inhibited HIV-1 replication in primary placental macrophages, possibly explaining why AZT can inhibit maternal fetal transmission in the absence of diminution of viral load [6].

Currently, viral load is used in conjunction with other parameters (e.g., CD4 counts, drug resistance genotyping, therapy history, appearance of side effects) to decide whether to initiate or modify anti-viral therapy. The observations that lower HIV-1 viral load may occur in HIV-1 positive women prompt the concern that their admission to anti-retroviral therapy under standard protocols could be inappropriately delayed, resulting in suboptimal efficacy in female patients. Consequently, it is important to systematically determine the effects of sex steroid hormones on HIV-1 replication, anti-retroviral drugs and combinations of hormones and anti-retroviral drugs. Here we ask whether the sex steroid hormone β-estradiol influences the efficacy of the anti-HIV drug, Stavudine (D4T).

## Results

### Hormone effect on anti-retroviral drugs in HIV-1 infection of PBL

2 nM β-estradiol depressed viral replication by ~26%. Although D4T was titered to achieve ~50% inhibition in preliminary experiments (not shown), when averaged over 8 experiments, the estimated "half-maximal" D4T concentration of about 50 nM resulted in an average reduced viral replication to 33% of virus alone (VA, Table 1). In 8 of the 8 experiments summarized in Tables 1 &2, virus levels in the presence of 2 nM β-estradiol in combination with 50 nM D4T were higher than in the presence of 50 nM D4T alone (individual experiments not shown). From the baseline average of 33% (of "VA") replication in 50 nM D4T, 2 nM β-estradiol increased HIV-1 replication in the presence of D4T to 74% (of VA, SE = 5.4), for a difference of 41% (of VA).

**Table 1:** Effects of 2 nM β-estradiol on HIV replication in the presence and absence of 50 nM D4T*

	Mean normalized pg/ml (%)**	Standard Error
virus alone (VA)	100	5.4
β-estradiol	74	5.4
D4T	33	5.4
D4T+β-estradiol	74	5.4
no virus control	3.9	6.9

*Data presented represents the combined results of 8 independent experiments on PBL from 8 different donors. For each experiment all data points were the averages of three cultures wells run in triplicate. Virus replication was assessed by p24Ag ELISA of culture supernatants.

**Data is presented as the mean of least squares normalized to "virus alone."

**Table 2:** Statistical Significance of Observed Differences*

	Δ**	Standard Error	T value	p***
"β-estradiol" – "virus alone"	-25.9	7.6	-3.4	0.0009
"D4T + β-estradiol" – "D4T alone"	41	7.6	5.4	< 0.0001

*Analysis of the dataset summarized in Table 1.

**Difference in raw percentages of data normalized to "virus alone."

***p = probability that the difference is zero.

**Figure 1:**
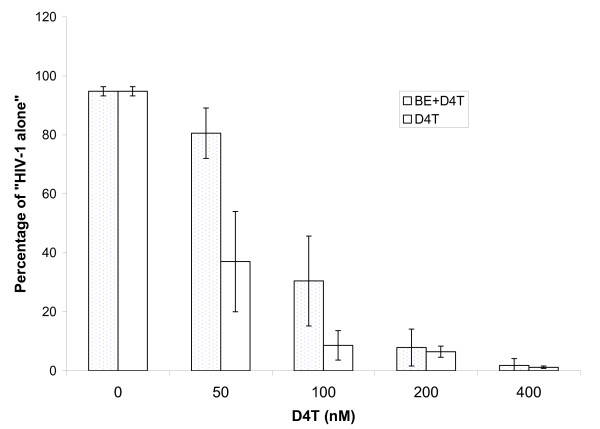
**Change in D4T concentration required to overcome the efficacy impairment caused by β-Estradiol**. A series of D4T dilutions were applied to HIV-1 infected PBL with or without 2 nM β-estradiol. The viral concentrations were measured with p24 ELISA, then normalized to the viral concentration from HIV-1 alone control. The data presented represents averages and standard deviations (error bars) from experiments performed on 4 different donors, each run in duplicate. Stippled, β-estradiol +D4T; clear, D4T alone.

To determine how the observed inhibition of drug efficacy translates into increased drug levels required to achieve half maximal virus inhibition in the presence of hormone, D4T was titered in the presence of 2 nM β-estradiol. In the presence of β-estradiol, an approximate 2 fold increase in D4T concentration is required to inhibit HIV-1 replication to levels seen in the absence of β-estradiol (compare results for 50 nM D4T only to 100 nM D4T + β-estradiol, Figure 1).

### Cell viability

To determine whether the observed effects were caused by non-specific effects on cell viability, cells were cultured without virus infection but with 2 nM β-estradiol alone or 2 nM β-estradiol plus 1 μM D4T under the conditions used for the experiments summarized in Tables 1 &2, and stained with trypan blue on day 7 of culture. The results show that the drugs and hormones were not toxic at the concentrations tested, as presented in Table 3, even though the concentration of D4T was over a log greater than the concentration used for the data presented in Tables 1 &2.

**Table 3:** Trypan blue resistance of PBL cultured with β-estradiol and/or D4T*

Treatments	Trypan blue negative cells as %*	
	
	Donor 1	Donor 2
No treatment	91	85
β-estradiol	88	83
β-estradiol + D4T	86	82
D4T	87	80

*Cells were prepared as if for infection and harvested after 7 days of culture in the concentrations of β-estradiol and/or D4T indicated. 200 cells total counted for each data point.

### Hormone concentration dependence of D4T efficacy

**Figure 2:**
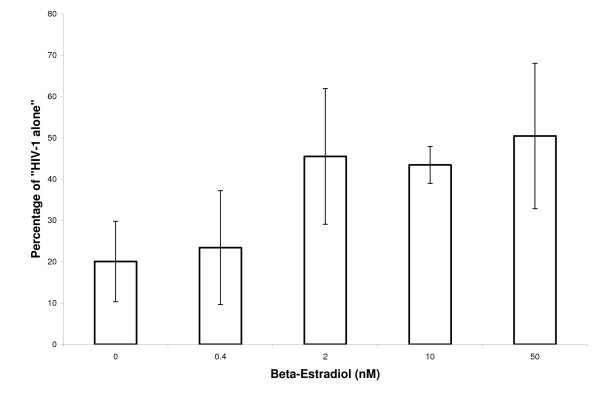
**β-estradiol concentration vs. D4T efficacy**. Serial dilutions of β-estradiol were combined with 100 nM D4T and then added to the HIV-1 infected PBL. The viral concentrations were measured with p24 ELISA, then normalized to the viral concentration from the "HIV-1 alone" control. Data was obtained from duplicate cultures from each of two different donors. Error bars represent the averaged results for each donor.

**Figure 3:**
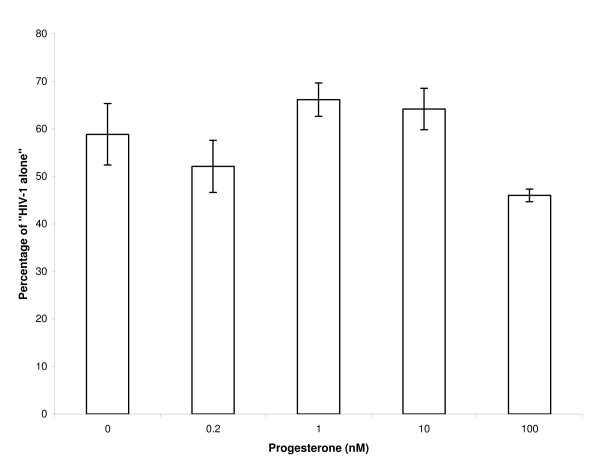
**Progesterone concentration vs. D4T efficacy**. Serial dilutions of progesterone were combined with 100 nM D4T and then added to the HIV-1 infected PBL. The viral concentrations were measured with p24 ELISA, then normalized to the viral concentration from the "HIV-1 alone" control. Data was obtained from duplicate cultures from each of two different donors. Error bars represent the averaged results for each donor.

Measurement of the effect of different concentrations of β-estradiol on D4T efficacy suggests that the reduction in efficacy titers over physiologically active levels of β-estradiol (Figure 2). For statistical analysis, the data were stratified into "no response" (0 & 0.4 nM β-estradiol and "response" (2, 10 & 50 nM β-estradiol) groups. The mean difference between response and no response in this experiment on 2 donors using 100 nM D4T was ~15 (percent of virus alone), which was statistically highly significant (p ≤ 0.0025). By way of contrast, similar titration of progesterone (from 0.2 nM to 100 nM) in the presence of D4T detected no effect of progesterone on D4T efficacy (Figure 3). Progesterone alone at these same concentrations also has no effect on HIV replication in PBL (data not shown).

**Figure 4:**
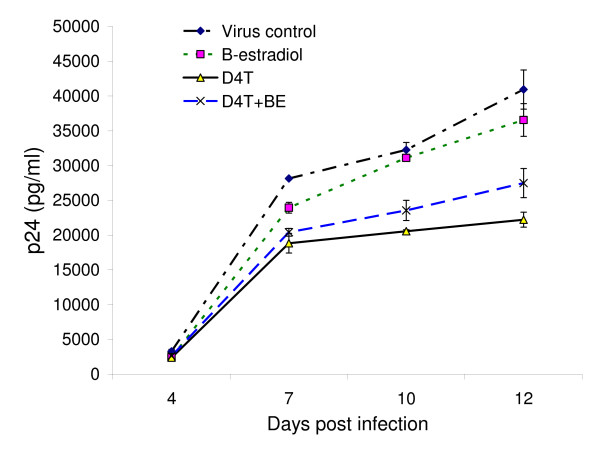
**Time course of viral replication in the presence or absence of D4T and/or β-estradiol**. HIV-infected PBL were cultured in the absence of drug and hormone, or in the presence of either 50 nM D4T or 2 nM β-estradiol, or the two together. Supernatants were harvested for p24 analysis on the indicated days. The data presented represent the results of infection of 2 different donors, with each arm run in triplicate wells of a 24-well plate containing 2 million PBL. Error bars represent standard deviations.

To determine the time course of the observed effects, HIV-infected PBL were cultured in the presence or absence of 50 nM D4T, 2 nM β-estradiol, or both together as above. Supernatant from the indicated days was analyzed for p24 Ag (Figure 4). The virus titers increased with time; however, the relative replication of virus in different arms remained the same at all of the time points tested after day 4, although the differences between each group became larger at later times.

**Figure 5:**
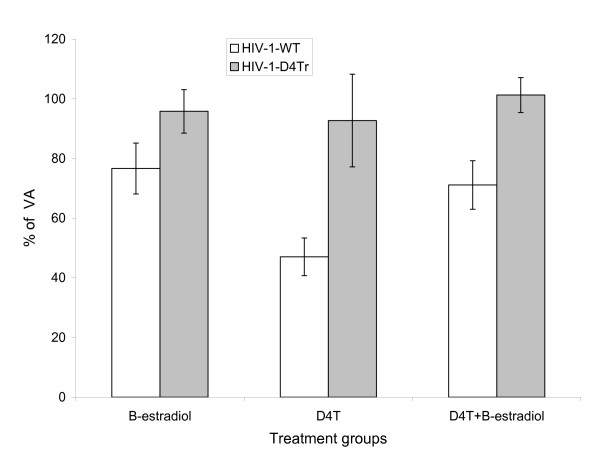
**The effect of D4T and β-Estradiol on the replication of a D4T-resistant mutant of HIV**. PBL were infected in parallel with wild type HIV-1 (strain 9320), open, or D4T-resistant HIV-1 (HIV-1-D4Tr), shaded, and were grown in the presence of either no added drug or hormone, 50 nM D4T alone, 2 nM β-estradiol alone, or the two together. Supernatants were harvested for p24 analysis on day 7. In each experiment infected cells were plated in triplicate wells of a 24 well plate, each containing 2 million PBL, and the average replication determined normalized to virus alone ("VA). The data presented represent the averages of three independent experiments on independent donors. The error bars represent +/- one SD.

To determine whether the observed effects involved a mechanism specific to the anti-reverse transcription action of D4T, we determined the responses of a D4T-resistant HIV mutant (HIV-1-D4Tr) to 50 nM D4T and 2 nM β-estradiol, separately and in combination. As seen in Figure 5, in the absence of D4T susceptibility, the enhancement effect of β-estradiol in the presence of D4T is abolished.

## Discussion

Interestingly, although β-estradiol modestly inhibits HIV-1 replication in PBL, it increases HIV-1 replication in the presence of a fixed amount of D4T, and this increase is specifically dependent on the anti-retroviral effect of the drug. Thus β-estradiol seems to decrease the efficacy of D4T against HIV-1 infection of PBL. The data suggests that the magnitude of the effect on D4T efficacy is such that approximately at least a two fold increase in the concentration of D4T would be necessary to overcome the effects of the hormone.

β-estradiol increased the amount of HIV-1 replication in the presence of D4T from a baseline of 33% (of VA, +/- SE = 5.4) to 74% (+/- SE = 5.4), (Tables 1 &2, Figure 1 & Figure 2) whereas progesterone had little or no effect on viral replication in the presence (or absence) of D4T (Figure 3). The concentrations of D4T used here for viral inhibition are within range of levels typically used for tissue culture work [7-10] and have not been reported to cause significant cytotoxicity. Nevertheless, we did test to see if the combinations of drugs and hormones studied caused detectable non-specific cytotoxicity in PBL. Even excessively high concentrations of D4T (1 uM) caused no cytotoxicity in the presence or absence of β-estradiol, as measured by trypan blue exclusion. β-estradiol alone also caused no cytotoxicity (Table 2).

The mechanism by which β-estradiol promotes HIV replication in the presence of D4T remains unknown. However, we have observed β-estradiol has no (or minimal) effect on HIV replication in the presence of the protease inhibitor, Saquinavir (unpublished observations). The finding that in the absence of D4T β-estradiol inhibits HIV replication, whereas in the presence of D4T it enhances HIV replication, strongly suggests that the mechanism of the enhancement is D4T-specific. In confirmation of this, we determined that β-estradiol has no effect on HIV replication in the presence of D4T when the HIV is resistant to D4T. Thus, the observed enhancement is most likely on the anti-retroviral efficacy of D4T. This is consistent with β-estradiol inhibiting the concentration or activity of the cellular enzymes used to phosphorylate D4T to its active form, D4T-TP, but does not rule out a mechanism involving changes in drug influx or efflux. Experiments to address these issues are currently ongoing in our laboratory.

The inhibition of antiretroviral drug efficacy by estrogen may have implications for anti-HIV-1 drug therapies. The studies presented here put forth the novel concept that at any given plasma concentration of drug, the final efficacy may be significantly affected by the hormone status of the patient. Most likely, β-estradiol acts by modifying intracellular levels of the active form of D4T through mechanisms which may include controlling drug influx or efflux or, more likely, controlling the phosphorylation steps which lead to the D4T-TTP active form of D4T [11,12]. Thus, monitoring of β-estradiol levels, which vary during pregnancy, menstrual cycling and with hormone replacement therapy and birth control, or monitoring intracellular levels of active drug may provide significant added benefit over monitoring plasma levels of drug alone.

## Methods

### Cell Culture

PBMC were isolated from the peripheral blood of HIV sero-negative donors (NIH Blood Bank) by Ficoll/Hypaque density gradient centrifugation. After monocytes were removed by adherence to the culture flasks, the remaining cells, PBL, were stimulated with 2 μg/ml PHA for 3 days to activate T cells before infection and either used fresh, or stored in liquid nitrogen before infection. The PBL cultures were maintained in 5% CO2 in complete RPMI (phenol red free RPMI 1640, supplemented with 10% heat-inactivated, charcoal dextran stripped FBS (Invitrogen, CA), 2 mM glutamine, 100 unit of penicillin per ml, 100 ug of streptomycin per ml, 10 mM HEPES), and 5 half-maximal units per ml of human Interleukin-2 (Roche, NJ).

### Virus

Wild type HIV was strain 9320, a low passage, AZT-sensitive isolate (A018, D. Richman) cultured in PBMC [13]. A highly AZT resistant strain of HIV-1 was obtained through the AIDS Research and Reference Reagent Program (Germantown, MD), AZT Resistant HIV-1 (catalogue number 629). We determined that this strain was cross resistant to D4T and referred to it in the text as "HIV-1-D4Tr." Stocks of HIV-1/9320 and HIV-1-D4Tr (both of which are known to replicate in PBL) were normalized to p24 Ag and tested for their ability to replicate in cultures of primary human microphages, in parallel with HIV-1 BAL (positive control) and HIV-1 IIIB (negative control). Both HIV-1/9320 and HIV-1-D4Tr strains replicated no better than HIV-1 IIIB in macrophages (data not shown).

### Hormones and anti-retroviral drugs

β-estradiol and progesterone and D4T were obtained from Sigma (St. Louis, MO). D4T was obtained from the NIH AIDS Research and Reference Reagent Program (Germantown, MD).

### Measurement of Virus Replication

Activated PBL cells were exposed to HIV strain 9320 or HIV-1-D4Tr for 2 hrs at 37°C at a concentration of 100 TCID50. Cells were then distributed into the appropriate arms of the experiment and cultured in triplicate wells of a 96 well plate at a concentration of 500,000 cells per well or a 24 well plate at a concentration of 2,000,000 cells per well in the same medium as for PBL culture. Cell supernatants were harvested on day 7 for analysis by HIV p24 ELISA (Perkin Elmer, MA). Each well was measured in singlicate. Similar results were obtained whether or not 50% of the supernatant of each well (including appropriate hormone/drug concentrations) were replaced on day 3–4.

### Statistics

Since the data were correlated, the PROC GLM in the SAS system was utilized to conduct the ANOVA analyses of the data while taking into consideration of the intra-correlation structure of the data. For the data presented in Tables 1 &2, each observation was translated into pg units based on the standard curve of the OD and pg reading. Then they were normalized into percentages of the "virus alone" data based on the average of the virus alone data for that particular experiment. Each of the 8 experiments was run in triplicate. A similar approach was used for the data in Figure 2, except that only two separate donors (experiments) were tested and they were tested in duplicate.

## Competing interests

The authors declare that they have no competing interests.

## Authors' contributions

MZ, QH, YH, OW, CC, and SD performed the experiments. WY performed the statistical analysis. All authors participated in the experimental design, data interpretation, and writing of the manuscript.
